# Clinical benefit of glasdegib plus low-dose cytarabine in patients with de novo and secondary acute myeloid leukemia: long-term analysis of a phase II randomized trial

**DOI:** 10.1007/s00277-021-04465-4

**Published:** 2021-03-19

**Authors:** Michael Heuser, B. Douglas Smith, Walter Fiedler, Mikkael A. Sekeres, Pau Montesinos, Brian Leber, Akil Merchant, Cristina Papayannidis, José A. Pérez-Simón, Caroline J. Hoang, Thomas O’Brien, Weidong Wendy Ma, Mirjana Zeremski, Ashleigh O’Connell, Geoffrey Chan, Jorge E. Cortes

**Affiliations:** 1 Department of Hematology, Hemostasis, Oncology and Stem Cell Transplantation, Hannover Medical School, Carl-Neuberg-Str. 1, 30625 Hannover, Germany; 2 Johns Hopkins Sidney Kimmel Comprehensive Cancer Center, Baltimore, MD USA; 3 Department of Hematology and Oncology, University Hospital Hamburg-Eppendorf, Hamburg, Germany; 4 Division of Hematology, Sylvester Comprehensive Cancer Center, University of Miami, FL Miami, USA; 5 Hospital Universitari i Politècnic La Fe, Valencia, Spain; 6 CIBERONC, Instituto Carlos III, Madrid, Spain; 7 Juravinski Hospital at Hamilton Health Sciences, Hamilton, ON Canada; 8 Samuel Oschin Comprehensive Cancer Institute, Cedars-Sinai Medical Center, Los Angeles, CA USA; 9 IRCCS Azienda Ospedaliero-Universitaria di Bologna, Bologna, Italy; 10 Hospital Universitario Virgen del Rocío, Instituto de Biomedicina (IbiS)/CSIC/CIBERONC), Universidad de Sevilla, Seville, Spain; 11 Pfizer Inc, New York, NY USA; 12 University of Texas MD Anderson Cancer Center, Houston, TX USA; 13 Present Address: Georgia Cancer Center, Augusta, GA USA

**Keywords:** Acute myeloid leukemia, Clinical trial, Glasdegib, Secondary acute myeloid leukemia

## Abstract

This analysis from the phase II BRIGHT AML 1003 trial reports the long-term efficacy and safety of glasdegib + low-dose cytarabine (LDAC) in patients with acute myeloid leukemia ineligible for intensive chemotherapy. The multicenter, open-label study randomized (2:1) patients to receive glasdegib + LDAC (de novo, *n* = 38; secondary acute myeloid leukemia, *n* = 40) or LDAC alone (de novo, *n* = 18; secondary acute myeloid leukemia, *n* = 20). At the time of analysis, 90% of patients had died, with the longest follow-up since randomization 36 months. The combination of glasdegib and LDAC conferred superior overall survival (OS) versus LDAC alone; hazard ratio (HR) 0.495; (95% confidence interval [CI] 0.325–0.752); *p* = 0.0004; median OS was 8.3 versus 4.3 months. Improvement in OS was consistent across cytogenetic risk groups. In a post-hoc subgroup analysis, a survival trend with glasdegib + LDAC was observed in patients with de novo acute myeloid leukemia (HR 0.720; 95% CI 0.395–1.312; *p* = 0.14; median OS 6.6 vs 4.3 months) and secondary acute myeloid leukemia (HR 0.287; 95% CI 0.151–0.548; *p* < 0.0001; median OS 9.1 vs 4.1 months). The incidence of adverse events in the glasdegib + LDAC arm decreased after 90 days’ therapy: 83.7% versus 98.7% during the first 90 days. Glasdegib + LDAC versus LDAC alone continued to demonstrate superior OS in patients with acute myeloid leukemia; the clinical benefit with glasdegib + LDAC was particularly prominent in patients with secondary acute myeloid leukemia. ClinicalTrials.gov identifier: NCT01546038.

**Supplementary Information:**

The online version contains supplementary material available at 10.1007/s00277-021-04465-4.

## Introduction

Acute myeloid leukemia (AML) is the most common type of leukemia in adults [1]. Secondary AML is associated with lower response rates and decreased overall survival (OS) with standard chemotherapy compared with de novo AML [2–6]. Although most cases arise de novo, AML can evolve from an antecedent myeloid disease or as a late complication from chemotherapy or ionizing radiation. Secondary AML accounts for approximately 25% of all AML cases (18–20% from a previous myeloid disease; 6–8% related to therapy) and occurs more frequently with increasing age [5, 7]. The incidence of secondary AML is highest between the ages of 70 and 79 years [7, 8]. Few randomized trials have been conducted that report data by diagnosis of de novo and secondary AML.

Induction with 7 + 3 chemotherapy has long remained the conventional treatment for patients with AML. In older adults receiving 7 + 3 regimens, induction mortality rates are higher, and response rates are lower than in younger individuals [6, 9]. Outcomes for older adults with secondary AML are particularly poor with 7 + 3 regimens. A recent phase III clinical trial demonstrated that CPX-351 (liposomal cytarabine and daunorubicin) significantly improved OS in patients aged ≥ 60 years with secondary AML versus standard 7 + 3 induction chemotherapy (9.56 vs 5.95 months, *p* = 0.0005) [10]. However, older patients with AML are often ineligible for such intensive chemotherapy because of comorbidities, performance status, or disease-related chemoresistance; therefore, they are treated with less-aggressive therapies including low-dose cytarabine (LDAC) and hypomethylating agents (HMAs; decitabine or azacitidine) [11, 12]. Studies with LDAC, decitabine, and azacitidine in older patients with AML have demonstrated median OS rates of 5 months, 7.7 months, and 10.4 months, respectively, indicating the need for novel therapeutic strategies to improve survival [13–15]. Several novel agents have recently been approved in the USA for the treatment of patients ineligible for intensive chemotherapy (e.g., glasdegib and venetoclax) [16, 17].

Glasdegib is a potent, selective, oral inhibitor of the Hedgehog signaling pathway. In a phase II randomized study that included patients with newly diagnosed AML or high-risk myelodysplastic syndromes (MDS) who were ineligible for intensive chemotherapy, the addition of glasdegib to LDAC demonstrated superior OS versus LDAC alone [18]. Based on the primary analysis of BRIGHT AML 1003, glasdegib was approved in the USA and Europe in combination with LDAC for the treatment of patients with newly diagnosed AML who are unable to receive intensive chemotherapy as a result of comorbidities or older age (≥ 75 years) [19, 20].

Here we report the efficacy and safety of glasdegib + LDAC in patients with AML after approximately 20 additional months of follow-up from the primary completion date. Exploratory analyses assessing the clinical benefit and safety of glasdegib + LDAC in AML subgroups based on de novo or secondary disease status are also reported.

## Methods

### Study design and patients

BRIGHT AML 1003 was an open-label, randomized, multicenter, phase II study (ClinicalTrials.gov identifier: NCT01546038) for which the methods have previously been published [18]. Briefly, BRIGHT AML 1003 enrolled adult patients aged ≥ 55 years with newly diagnosed, previously untreated AML or high-risk MDS (World Health Organization 2008 classification), who were ineligible for intensive chemotherapy, defined as meeting ≥ 1 of the following criteria: ≥ 75 years old; severe cardiac disease; baseline Eastern Cooperative Oncology Group performance status (ECOG PS) = 2; or baseline serum creatinine > 1.3 mg/dL [19]. Glasdegib 100 mg was administered orally, once daily, on a continuous basis. LDAC 20 mg was administered subcutaneously, twice daily for 10 days, every 28 days. Treatments continued until disease progression, unacceptable toxicity, or patient refusal. This long-term analysis assessed efficacy and safety in patients with AML only.

The study was conducted in accordance with the Declaration of Helsinki. All patients provided written informed consent before study procedures began, and the protocol was approved by institutional review boards at each study site.

### Efficacy and safety assessments

The study’s primary endpoint was OS. The secondary endpoint of response to treatment was assessed based on the International Working Group response criteria for AML [21].

Transfusion rates and recovery of the three blood cell lineages, all at two thresholds, were also measured: absolute neutrophil count (≥ 1000/μL or 500/μL); hemoglobin (≥ 10 g/dL or 9 g/dL); and platelets (≥ 100,000/μL or 50,000/μL). Time to recovery and treatment cycle analyses included only remaining patients at risk in that cycle. Transfusion independence was defined as ≥ 8 weeks without transfusions.

Safety assessments included adverse events (AEs), classified and graded based on the National Cancer Institute Common Terminology Criteria for Adverse Events v4.0, laboratory evaluations, vital signs, physical examinations, and 12-lead electrocardiograms.

A post-hoc subgroup analysis was also performed in patients categorized by diagnosis as determined by the investigator: de novo AML or secondary AML. Secondary AML was defined as AML evolving from MDS or other antecedent hematologic disease, or AML after previous cytotoxic therapy or radiation.

### Biomarker analysis

Biomarker assessments included determination of baseline mutational status of the following genes: *CEBPA*, *DNMT3A*, *FLT3*, *IDH1*, *IDH2*, *KIT*, *KRAS*, *NPM1*, *NRAS*, *RUNX1*, *TET2*, and *WT1*.

### Statistical analyses

After discontinuation of study treatment, patients were followed until death or for 4 years from the first dose. OS was estimated using the Kaplan–Meier method, and 95% confidence intervals (CIs) of median OS were calculated using the Brookmeyer and Crowley method. For biomarker analyses, a Cox proportional hazard regression model was used to estimate the hazard ratio (HR) and 95% CI of OS using the non-mutated gene or the LDAC alone arm as the reference group. Exposure-adjusted transfusion rates were calculated as the sum of on-study transfusions/total time on treatment for all patients in the treatment arm. Other efficacy endpoints were summarized descriptively. Safety data were also summarized descriptively and included all randomized patients who received at least one dose of any of the study medications.

## Results

### Disposition, demography, and baseline characteristics

One hundred and sixteen patients with AML were randomized (2:1) to treatment with glasdegib + LDAC (de novo, *n* = 38; secondary AML, *n* = 40) or LDAC alone (de novo, *n* = 18; secondary AML, *n* = 20); among them, 75 (de novo, *n* = 38; secondary AML, *n* = 37) and 36 (de novo, *n* = 17; secondary AML, *n* = 19) patients received study treatments, respectively (Online Resource, Fig. S1). At the time of data cut-off (11 October 2018), three patients in the glasdegib + LDAC arm (de novo, *n* = 1; secondary AML, *n* = 2) had received ≥ 172 weeks of treatment and were still receiving therapy. The most common reason for treatment discontinuation among all patient cohorts was insufficient clinical response.

Baseline demographics and disease characteristics were generally similar across the two treatment arms (Table 1). At study entry, the median age was 77 years (range, 64–92) in the glasdegib + LDAC arm and 76 years (range, 58–83) in the LDAC arm; 51.3% and 52.6% of patients had a diagnosis of secondary AML, respectively. In the overall population (de novo + secondary AML), the median duration of treatment was 83.0 days (range, 3–1492) and 40.5 days (range, 6–239) in the glasdegib + LDAC and LDAC alone arms, respectively; 13/75 patients (17.3%) were treated with glasdegib + LDAC for at least 1 year (Online Resource, Table S1). The median duration of treatment for glasdegib + LDAC and LDAC alone, respectively, was 69.5 days (range, 5–1206) and 47.0 days (range, 10–239) in the de novo AML subgroup and 101.0 days (range, 3–1492) and 39.0 days (range, 6–149) in the secondary AML subgroup.

**Table 1:** Demography and baseline characteristics by diagnosis of AML

Characteristic	Overall population		de novo AML		Secondary AML	
	Glasdegib + LDAC *n* = 78	LDAC alone *n* = 38	Glasdegib + LDAC *n* = 38	LDAC alone *n* = 18	Glasdegib + LDAC *n* = 40	LDAC alone *n* = 20
Sex, *n* (%)						
Female	19 (24.4)	15 (39.5)	10 (26.3)	8 (44.4)	9 (22.5)	7 (35.0)
Male	59 (75.6)	23 (60.5)	28 (73.7)	10 (55.6)	31 (77.5)	13 (65.0)
Age, years, *n* (%)						
Mean (SD)	76.4 (6.0)	74.8 (4.9)	76.6 (5.8)	74.6 (5.1)	76.2 (6.3)	74.9 (4.9)
Median (range)	77.0 (64–92)	76.0 (58–83)	77.0 (64–87)	75.5 (58–80)	76.0 (65–92)	76.0 (65–83)
Secondary AML, *n* (%)						
Prior hematologic disease	34 (43.6)	19 (50.0)	–	–	34 (85.0)	19 (95.0)
MDS	29 (37.2)	15 (39.5)	–	–	29 (72.5)	15 (75.0)
Other	5 (6.4)	4 (10.5)	–	–	5 (12.5)	4 (20.0)
Chemotherapy/ radiotherapy	6 (7.7)	1 (2.6)	–	–	6 (15.0)	1 (5.0)
Prior therapy with MDS drug, *n* (%)						
Azacitidine	10 (12.8)	5 (13.2)	–	–	10 (25.0)	5 (25.0)
Decitabine	1 (1.3)	1 (2.6)	–	–	1 (2.5)	1 (5.0)
Duration since diagnosis, median, months	0.6	0.5	0.5	0.5	0.6	0.6
First-line AML non-intensive population criteria, *n* (%)						
Age ≥ 75 years	48 (61.5)	23 (60.5)	25 (65.8)	12 (66.7)	23 (57.5)	11 (55.0)
ECOG PS = 2	41 (52.6)	18 (47.4)	18 (47.4)	8 (44.4)	23 (57.5)	10 (50.0)
sCr > 1.3 mg/dL	15 (19.2)	5 (13.2)	7 (18.4)	4 (22.2)	8 (20.0)	1 (5.0)
Severe cardiac disease	52 (66.7)	20 (52.6)	29 (76.3)	9 (50.0)	23 (57.5)	11 (55.0)
Cytogenetic risk, *n* (%)^a^						
Good/intermediate	53 (67.9)	22 (57.9)	25 (65.8)	14 (77.8)	28 (70.0)	8 (40.0)
Poor	25 (32.1)	16 (42.1)	13 (34.2)	4 (22.2)	12 (30.0)	12 (60.0)
ELN risk stratification for AML, *n* (%) [22]						
Favorable	5 (6.4)	3 (7.9)	3 (7.9)	2 (11.1)	2 (5.0)	1 (5.0)
Intermediate I	27 (34.6)	11 (28.9)	13 (34.2)	5 (27.8)	14 (35.0)	6 (30.0)
Intermediate II	21 (26.9)	8 (21.1)	9 (23.7)	7 (38.9)	12 (30.0)	1 (5.0)
Adverse	25 (32.1)	16 (42.1)	13 (34.2)	4 (22.2)	12 (30.0)	12 (60.0)
Mutations, *n* (%)^b^						
*FLT3*	5 (6.4)	0	3 (7.9)	0	2 (5.0)	0
*IDH1* or *IDH2*	19 (24.3)	6 (15.8)	14 (29.2)	2 (11.1)	5 (12.5)	4 (20.0)
*NPM1*	5 (6.4)	1 (2.6)	3 (7.9)	1 (5.6)	2 (5.0)	0
White blood cell count (10^3^/mm^3^)						
Median (range)	2.7 (0.4–5850.0)	3.8 (1.2–1370.0)	2.7 (0.4–28.0)	4.0 (1.6–27.9)	2.7 (0.5–5850.0)	3.7 (1.2–1370.0)
Hemoglobin (g/dL)						
Median (range)	8.7 (6.9–13.8)	9.0 (6.9–13.4)	8.8 (6.9–13.8)	9.2 (7.4–12.4)	8.7 (7.3–12.2)	8.9 (6.9–13.4)
Platelet count (10^3^/mm^3^)						
Median (range)	42.0 (7.0–35,000.0)	26.5 (3.0–23,000.0)	55.0 (10.0–258.0)	34.0 (11.0–199.0)	30.0 (7.0–35,000.0)	22.0 (3.0–23,000.0)
Bone marrow blasts, %						
Median (range)	41.0 (16.0–99.0)	46.0 (13.0–95.0)	47.5 (20.8–99.0)	50.5 (20.0–87.0)	38.0 (16.0–95.0)	43.0 (13.0–95.0)

*AML*, acute myeloid leukemia; *ECOG PS*, Eastern Cooperative Oncology Group performance status; *ELN*, European LeukemiaNet; *LDAC,* low-dose cytarabine; *MDS*, myelodysplastic syndromes; *sCr*, serum creatinine; *SD*, standard deviation

^a^For AML, good/intermediate cytogenetic risk = favorable, Intermediate I and Intermediate II risk groups; poor cytogenetic risk = adverse risk group

^b^Baseline gene mutations were determined in 58/78 glasdegib/LDAC patients (de novo AML, *n* = 30; secondary AML, *n* = 28) and 25/38 LDAC alone patients (de novo AML, *n* = 11; secondary AML, *n* = 14)

Among patients who received treatment, 34/75 (45.3%) in the glasdegib + LDAC arm and 11/36 (30.6%) in the LDAC alone arm were reported to have received subsequent systemic therapies after discontinuation of the study treatment. The most frequent subsequent therapy was HMAs in 52.9% (18/34) and 36.4% (4/11) of patients in the glasdegib + LDAC and LDAC alone arm, respectively.

In the overall AML population, at the time of analysis, 90% of patients had died, with the longest follow-up time since randomization 36 months.

### Efficacy

**Fig. 1:**
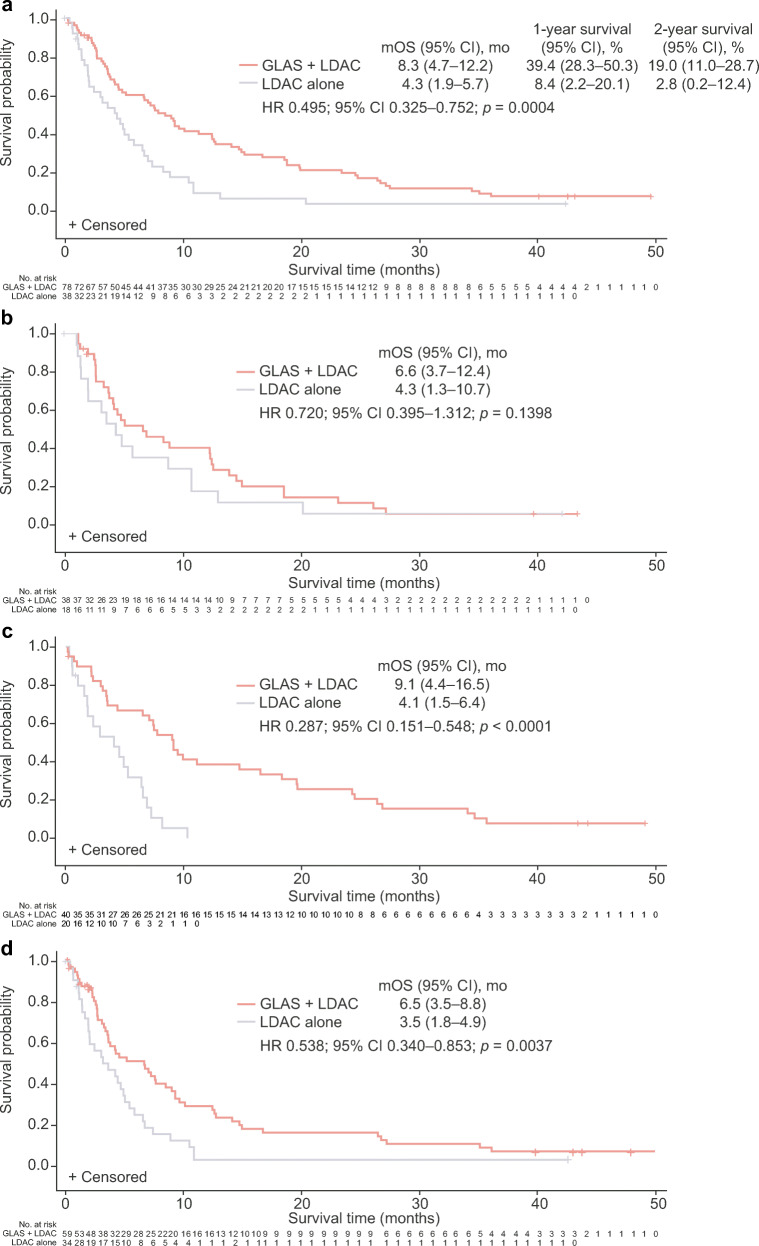
Kaplan–Meier plots of overall survival in the **a** overall population, **b** de novo AML subgroup, **c** secondary AML subgroup, and **d** overall population censoring for patients receiving follow-up HMAs. GLAS, glasdegib; mo, months; mOS, median overall survival

In the overall AML population, treatment with glasdegib + LDAC resulted in superior OS versus LDAC alone (HR 0.495; 95% CI 0.325–0.752; *p* = 0.0004); median OS was 8.3 (95% CI 4.7–12.2) versus 4.3 (95% CI 1.9–5.7) months (Fig. 1a and Table 2). The respective survival probability was 39.4% (95% CI 28.3–50.3) and 8.4% (95% CI 2.2–20.1) at 1 year, and 19.0% (95% CI 11.0–28.7) and 2.8% (95% CI 0.2–12.4) at 2 years. The improvement in survival with glasdegib + LDAC was consistent across most groups stratified by cytogenetic risk (Table 2).

**Table 2:** Overall survival in patients with AML

	Overall population		de novo AML		Secondary AML	
	Glasdegib + LDAC	LDAC alone	Glasdegib + LDAC	LDAC alone	Glasdegib + LDAC	LDAC alone
**All patients**	***n*** **= 78**	***n*** **= 38**	***n*** **= 38**	***n*** **= 18**	***n*** **= 40**	***n*** **= 20**
Median OS, months (95% CI)	8.3 (4.7–12.2)	4.3 (1.9–5.7)	6.6 (3.7–12.4)	4.3 (1.3–10.7)	9.1 (4.4–16.5)	4.1 (1.5–6.4)
HR (95% CI)	0.495 (0.325–0.752)		0.720 (0.395–1.312)		0.287 (0.151–0.548)	
*p* value	0.0004		0.1398		< 0.0001	
Deaths, *n* (%)						
Total	69 (88.5)	35 (92.1)	33 (86.8)	16 (88.9)	36 (90.0)	19 (95.0)
Cause of death: disease progression	59 (75.6)	29 (76.3)	28 (73.7)	12 (66.7)	31 (77.5)	17 (85.0)
**Good/intermediate cytogenetic risk,** ***n*** **(%)**^a^	**53 (67.9)**	**22 (57.9)**	**25 (65.8)**	**14 (77.8)**	**28 (70.0)**	**8 (40.0)**
Median OS, months (95% CI)	12.2 (6.9–16.5)	5.3 (3.5–8.7)	12.2 (3.7–14.9)	4.3 (1.3–10.7)	11.1 (6.5–24.4)	6.9 (4.1–8.1)
HR (95% CI)	0.510 (0.294–0.886)		0.603 (0.295–1.233)		0.350 (0.135–0.907)	
*p* value	0.0074		0.0792		0.0121	
Deaths, *n* (%)						
Total	45 (84.9)	19 (86.4)	21 (84.0)	12 (85.7)	24 (85.7)	7 (87.5)
Cause of death: disease progression	37 (69.8)	15 (68.2)	16 (64.0)	9 (64.3)	21 (75.0)	6 (75.0)
**Poor cytogenetic risk,** ***n*** **(%)**^b^	**25 (32.1)**	**16 (42.1)**	**13 (34.2)**	**4 (22.2)**	**12 (30.0)**	**12 (60.0)**
Median OS, months (95% CI)	4.4 (2.6–7.4)	2.1 (1.0–4.9)	4.1 (1.9–8.8)	4.4 (1.1–12.9)	5.7 (0.2–9.1)	1.8 (0.5–4.9)
HR (95% CI)	0.514 (0.264–1.000)		1.077 (0.337–3.441)		0.301 (0.109–0.829)	
*p* value	0.0229		0.5495		0.0073	
Deaths, *n* (%)						
Total	24 (96.0)	16 (100.0)	12 (92.3)	4 (100.0)	12 (100.0)	12 (100.0)
Cause of death: disease progression	22 (88.0)	14 (87.5)	12 (92.3)	3 (75.0)	10 (83.3)	11 (91.7)

*AML*, acute myeloid leukemia; *CI*, confidence interval; *HR*, hazard ratio; *LDAC*, low-dose cytarabine; *OS*, overall survival

^a^Favorable, Intermediate I and Intermediate II risk groups

^b^Adverse risk group

In the de novo AML subgroup, median OS was 6.6 (95% CI 3.7–12.4) months with glasdegib + LDAC, and 4.3 (95% CI 1.3–10.7) months with LDAC alone (HR 0.720; 95% CI 0.395–1.312; *p* = 0.1398) (Fig. 1b and Table 2). The respective survival probability was 40.4% (95% CI 24.3–55.9) and 17.6% (95% CI 4.3–38.3) at 1 year, and 11.5% (95% CI 3.7–24.4) and 5.9% (95% CI 0.4–23.5) at 2 years. The survival benefit of glasdegib + LDAC versus LDAC alone was more pronounced among patients in the secondary AML subgroup (HR 0.287; 95% CI 0.151–0.548; *p* < 0.0001); median OS was 9.1 (95% CI 4.4–16.5) months and 4.1 (95% CI 1.5–6.4) months, respectively (Fig. 1c). The survival probabilities at 1 and 2 years, respectively, were 38.5% (95% CI 23.6–53.3) and 25.7% (95% CI 13.3–39.9) with glasdegib + LDAC, and 0% with LDAC alone (all patients died within the first year).

Among patients in the overall population who received prior therapy with HMAs (glasdegib + LDAC, *n* = 11; LDAC alone, *n* = 6), the median OS was 7.1 (95% CI 2.2–14.7) months with glasdegib + LDAC and 5.1 (95% CI 0.5–7.2) months with LDAC alone (HR 0.438; 95% CI 0.138–1.391; *p* = 0.0754). Of the patients in the overall population without prior HMA therapy (glasdegib + LDAC, *n* = 67; LDAC alone, *n* = 32), the median OS was 8.8 (95% CI 4.7–12.4) months with glasdegib + LDAC and 4.1 (95% CI 1.8–6.4) months with LDAC alone (HR 0.500; 95% CI 0.317–0.789; *p* = 0.0012); among patients with secondary AML (glasdegib + LDAC, *n* = 29; LDAC alone, *n* = 14), the median OS was 9.9 (95% CI 6.5–19.6) months with glasdegib + LDAC and 2.9 (95% CI 1.0–6.4) months with LDAC alone. For patients who did not receive subsequent HMAs in the overall population, the median OS was 6.5 (95% CI 3.5–8.8) months with glasdegib + LDAC and 3.5 (95% CI 1.8–4.9) months with LDAC alone (Fig. 1d); among patients with secondary AML, the median OS was 7.4 (95% CI 3.1–9.9) months with glasdegib + LDAC and 2.9 (95% CI 1.5–5.3) months with LDAC alone.

**Fig. 2:**
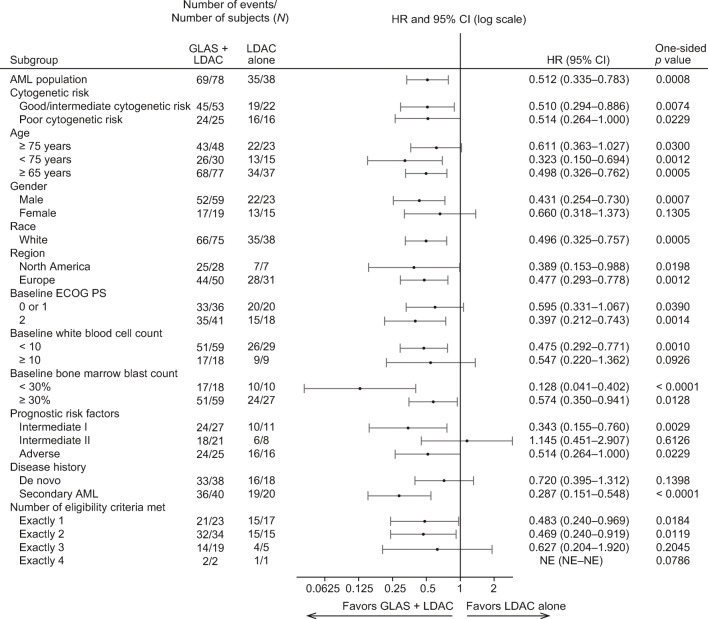
Forest plot of overall survival for patients with AML (overall population). GLAS, glasdegib; NE, not evaluable

To further define the survival benefit of glasdegib + LDAC, a subgroup analysis was performed by baseline characteristics. The improvement in OS with glasdegib + LDAC was consistent across most subgroups in the overall population (Fig. 2), and by the diagnosis of de novo and secondary AML (Online Resource, Fig. S2 and Online Resource, Fig. S3). Consistent with a benefit in secondary AML, patients with less than 30% blasts at diagnosis and patients who developed AML from a prior hematologic disease derived a survival benefit from glasdegib + LDAC.

In the overall AML population, 15 patients (19.2%) in the glasdegib + LDAC arm and one patient (2.6%) in the LDAC alone arm achieved complete remission (CR). The median duration of CR was 302 days in the glasdegib + LDAC arm and 91 days in the LDAC alone arm. The rates of CR were similar for patients with de novo (18.4%) and secondary (20.0%) AML in the glasdegib + LDAC arm; however, duration of remission was longer in patients with secondary AML (532 days) versus de novo AML (175 days) (Table 3). Among patients in the overall population who received prior therapy with HMAs, 9.1% (*n* = 1/11) of patients in the glasdegib + LDAC arm and no patients (*n* = 0/6) in the LDAC alone arm achieved CR; the median duration of CR was 64 days in the glasdegib + LDAC arm. Of the patients in the overall population without prior HMA therapy, 20.9% (*n* = 14/67) in the glasdegib + LDAC arm and 3.1% (*n* = 1/32) in the LDAC alone arm achieved CR; among patients with secondary AML, 24.1% (*n* = 7/29) in the glasdegib + LDAC arm and no patients (*n* = 0/14) in the LDAC alone arm achieved CR. In the overall population, the median duration of CR was 379.5 (95% CI 1–1262) days with glasdegib + LDAC and 91 days with LDAC alone; for patients with secondary AML receiving glasdegib + LDAC, the median duration of CR was 574.0 (95% CI 302–1262) days.

**Table 3:** CR in patients with AML

	Overall population		de novo AML		Secondary AML	
	Glasdegib + LDAC	LDAC alone	Glasdegib + LDAC	LDAC alone	Glasdegib + LDAC	LDAC alone
	*n* = 78	*n* = 38	*n* = 38	*n* = 18	*n* = 40	*n* = 20
Patients with CR, *n* (%)	15 (19.2)	1 (2.6)	7 (18.4)	1 (5.6)	8 (20.0)^b^	0
95% CI^a^	10.5–28.0	0.0–7.7	6.1–30.7	0.0–16.1	7.6–32.4	N/A
Duration of remission, days						
Median (range)	302 (1–1262)	91 (91–91)	175 (1–533)	91 (91–91)	532 (64–1262)	0
Cytogenetic risk						
**Good/intermediate risk**	***n*** **= 49**	***n*** **= 21**	***n*** **= 24**	***n*** **= 14**	***n*** **= 25**	***n*** **= 7**
Patients with CR, *n* (%)	11 (22.4)	0	5 (20.8)	0	6 (24.0)	0
95% exact CI^c^	11.8–36.6	0.0–16.1	7.1–42.2	0.0–23.2	9.4–45.1	0.0–41.0
**Poor cytogenetic risk**	***n*** **= 29**	***n*** **= 17**	***n*** **= 14**	***n*** **= 4**	***n*** **= 15**	***n*** **= 13**
Patients with CR, *n* (%)	4 (13.8)	1 (5.9)	2 (14.3)	1 (25.0)	2 (13.3)	0
95% exact CI^c^	3.9–31.7	0.1–28.7	1.8–42.8	0.6–80.6	1.7–40.5	0.0–24.7
Combination vs LDAC						
Pearson Chi-square test (unstratified), *p* value	0.0150		0.1988		0.0317	
CMH test stratified by CRF prognosis factor						
Odds ratio (95% CI)	4.3762 (1.3296–14.4037)		1.8306 (0.4931–6.7962)		4.5370 (1.0712–19.2173)	
*p* value	0.0184		0.2207		0.0662	

*AML*, acute myeloid leukemia; *CI*, confidence interval; *CMH*, Cochran–Mantel–Haenszel; *CR*, complete remission; *CRF*, case report form; *LDAC*, low-dose cytarabine; *MDS*, myelodysplastic syndrome; *N/A*, not applicable

^a^Using normal approximation

^b^Secondary AML developed from prior MDS (*n* = 7) or from prior chemotherapy/radiotherapy treatment (*n* = 1)

^c^Using exact method based on binomial distribution

zIn the overall AML population, more patients achieved durable (≥ 2 consecutive assessments) recovery of absolute neutrophil count, hemoglobin, and platelets in the glasdegib + LDAC arm than in the LDAC alone arm (Table 4 and Online Resource, Fig. S4). The median time to recovery with glasdegib + LDAC versus LDAC alone was longer for absolute neutrophil count (≥ 1000/μL, 27 vs 13 days; ≥ 500/μL, 16 vs 11 days), shorter for hemoglobin (≥ 10 g/dL, 22 vs 33 days; ≥ 9 g/dL, 14 vs 22 days), and similar for platelets (≥ 100,000/μL, 30 vs 26 days; ≥ 50,000/μL, 26 vs 24 days). Patients in the glasdegib + LDAC arm had fewer transfusions than those receiving LDAC alone; the difference was more significant in favor of patients receiving glasdegib + LDAC when adjusted for duration of treatment (Table 4). In the overall population, transfusion independence was achieved by 29.3% of patients receiving glasdegib + LDAC and 5.6% of patients receiving LDAC alone; the median duration of independence was 212 days (range, 56–1054) and 144 days (range, 141–147), respectively. Bone marrow recovery and transfusion independence occurred at similar rates in the de novo and secondary AML subgroups (Online Resource, Table S2).

**Table 4:** Recovery of ANC, hemoglobin, and platelets, and rates of transfusions in patients with AML (overall population)

	Glasdegib + LDAC	LDAC alone	Glasdegib + LDAC	LDAC alone
	*n* = 72	*n* = 32	*n* = 72	*n* = 32
**ANC**	**≥ 1000/μL**		**≥ 500/μL**	
All patients with recovery, *n* (%)	49 (68.1)	20 (62.5)	58 (80.6)	24 (57.1)
Recovery at ≥ 2 consecutive visits, *n* (%)	40 (55.6)	12 (37.5)	47 (65.3)	17 (53.1)
Baseline ANC < threshold, *n* (%)^a^	22 (30.5)	5 (15.6)	18 (25.0)	1 (3.1)
Median time to first recovery, days (range)	27 (7–114)	13 (8–70)	16 (3–143)	11 (8–119)
Achieved recovery during cycle 2/1, *n* (%)^b^	30 (41.7)	10 (31.3)	49 (68.1)	22 (68.8)
**Hemoglobin**	**10 g/dL**		**≥ 9 g/dL**	
All patients with recovery, *n* (%)	43 (59.7)	18 (56.3)	64 (88.9)	22 (68.8)
Recovery at ≥2 consecutive visits, *n* (%)	23 (31.9)	7 (21.9)	44 (61.1)	13 (40.6)
Baseline hemoglobin < threshold, *n* (%)^a^	19 (26.4)	4 (12.5)	24 (33.3)	2 (6.3)
Median time to first recovery, days (range)	22 (6–129)	33 (9–140)	14 (4–172)	22 (2–85)
Achieved recovery during cycle 1, *n* (%)	31 (43.1)	11 (34.4)	57 (79.2)	20 (62.5)
**Platelets**	**≥ 100,000/μL**		**≥50,000/µL**	
All patients with recovery, *n* (%)	36 (50.0)	7 (21.9)	49 (68.1)	13 (40.6)
Recovery at ≥2 consecutive visits, *n* (%)	30 (41.7)	4 (12.5)	38 (52.8)	8 (25.5)
Baseline platelets < threshold, *n* (%)^a^	20 (27.8)	2 (6.25)	15 (20.8)	3 (9.4)
Median time to first recovery, days (range)	30 (6–171)	26 (2–56)	26 (4–141)	24 (2–119)
Achieved recovery during cycle 1, *n* (%)	24 (33.3)	6 (18.8)	40 (55.6)	11 (34.4)
**Transfusion rates**	**Glasdegib + LDAC** ***n*** **= 75**		**LDAC alon**e ***n*** **= 36**	
**Proportion independent,** *n*** (%)**^c^				
No transfusions	22 (29.3)		2 (5.6)	
PRBC transfusions	25 (33.3)		3 (8.3)	
Platelet transfusion	32 (42.7)		4 (11.1)	
**Exposure-adjusted rate**				
Any transfusion	0.0696		0.1555	
PRBC transfusion	0.0423		0.0789	
Platelet transfusion	0.0273		0.0766	

*AML*, acute myeloid leukemia; *ANC*, absolute neutrophil count*; LDAC*, low-dose cytarabine; *PRBC*, packed red blood cell

^a^Requires measurement at ≥ 2 consecutive visits

^b^Cycle 2 for ANC ≥ 1000/μL and cycle 1 for ANC ≥ 500/μL

^c^Required no PRBC or platelet transfusions for ≥ 8 weeks

### Safety

To account for imbalances in treatment duration between the glasdegib + LDAC (≤ 90 days, *n* = 75; > 90 days, *n* = 43) and LDAC alone (≤ 90 days, *n* = 36; > 90 days, *n* = 14) arms, AEs are presented separately for the first 90 days. For patients completing > 90 days of treatment, the median duration of treatment was 262.5 days (range, 97–1492) and 122.5 days (range, 94–239) in the glasdegib + LDAC and LDAC alone arms, respectively. After 90 days, the mean relative dose intensity for glasdegib was 89.4% for the glasdegib + LDAC arm, and the mean relative LDAC intensity was 97.5% and 97.4% for the glasdegib + LDAC and LDAC alone arms, respectively.

All of the patients randomized and treated in both arms reported treatment-emergent AEs during the course of the study. The incidence of AEs was lower over the long term (after 90 days) than the short term (during the first 90 days) in both the glasdegib + LDAC (≤ 90 days, 98.7%; > 90 days, 83.7%) and LDAC alone arms (≤ 90 days, 100.0%; > 90 days, 71.4%) (Table S3). In the glasdegib + LDAC arm, the most common treatment-emergent AEs over the short term and long term were anemia and diarrhea, respectively. Corresponding AEs in the LDAC alone arm were anemia and anemia/decreased appetite/pneumonia (Table 5). AEs thought to be linked to the inhibition of the Hedgehog signaling pathway in normal tissue occurred in patients receiving glasdegib + LDAC over the short term (alopecia, 4.0%; dysgeusia, 20.0%; muscle spasms, 14.7%) and long-term (alopecia, 9.3%; dysgeusia, 14.0%; muscle spasms, 23.3%). The proportion of patients who experienced grade 3/4 AEs was lower over the long term than the short term in both the glasdegib + LDAC (≤ 90 days, 84.0%; > 90 days, 69.8%) and LDAC alone arms (≤ 90 days, 91.7%; > 90 days, 57.1%). The severity and rate of AEs were similar in the de novo and secondary AML subgroups (Tables S3 and S4).

**Table 5:** Treatment-emergent all-causality AEs occurring in ≥ 20% of patients (overall population) in any treatment arm during the first 90 days and after 90 days of therapy

MedDRA preferred term, *n* (%)^a^	Glasdegib + LDAC			LDAC alone		
	Grade 3–4	Grade 5	All AEs	Grade 3–4	Grade 5	All AEs
**During the first 90 days**	***n*** **= 75**			***n*** **= 36**		
Any AEs	53 (70.7)	12 (16.0)	74 (98.7)	20 (55.6)	13 (36.1)	36 (100.0)
Anemia	31 (41.3)	0	33 (44.0)	13 (36.1)	0	15 (41.7)
Febrile neutropenia	23 (30.7)	0	23 (30.7)	8 (22.2)	0	8 (22.2)
Thrombocytopenia	23 (30.7)	0	23 (30.7)	8 (22.2)	0	9 (25.0)
Nausea	1 (1.3)	0	22 (29.3)	1 (2.8)	0	4 (11.1)
Fatigue	7 (9.3)	0	19 (25.3)	2 (5.6)	0	6 (16.7)
Edema peripheral	0	0	17 (22.7)	1 (2.8)	0	7 (19.4)
Constipation	1 (1.3)	0	15 (20.0)	0	0	5 (13.9)
Decreased appetite	0	0	15 (20.0)	1 (2.8)	0	3 (8.3)
Dysgeusia	0	0	15 (20.0)	0	0	1 (2.8)
Pyrexia	1 (1.3)	0	15 (20.0)	1 (2.8)	0	8 (22.2)
Vomiting	2 (2.7)	0	15 (20.0)	1 (2.8)	0	3 (8.3)
Pneumonia	8 (10.7)	3 (4.0)	14 (18.7)	7 (19.4)	1 (2.8)	9 (25.0)
Diarrhea	1 (1.3)	0	13 (17.3)	0	0	9 (25.0)
Dyspnea	4 (5.3)	0	13 (17.3)	2 (5.6)	0	9 (25.0)
**After 90 days**	***n*** **= 43**			***n*** **= 14**		
Any AEs	22 (51.2)	10 (23.3)	36 (83.7)	6 (42.9)	3 (21.4)	10 (71.4)
Diarrhea	3 (7.0)	0	14 (32.6)	1 (7.1)	0	1 (7.1)
Anemia	10 (23.3)	0	13 (30.2)	3 (21.4)	0	3 (21.4)
Decreased appetite	3 (7.0)	0	13 (30.2)	2 (14.3)	0	3 (21.4)
Muscle spasms	4 (9.3)	0	10 (23.3)	0	0	0
Pyrexia	1 (2.3)	0	10 (23.3)	1 (7.1)	0	1 (7.1)
Thrombocytopenia	9 (20.9)	0	10 (23.3)	2 (14.3)	0	2 (14.3)
Nausea	1 (2.3)	0	9 (20.9)	0	0	0
Neutropenia	7 (16.3)	0	9 (20.9)	1 (7.1)	0	1 (7.1)
Pneumonia	3 (7.0)	3 (7.0)	8 (18.6)	1 (7.1)	2 (14.3)	3 (21.4)

*AE*, adverse event; *LDAC*, low-dose cytarabine; *MedDRA*, Medical Dictionary for Regulatory Activities

^a^Patients are counted only once per preferred term in each row. Each count is based on the maximum grade of events

Serious AEs were reported in 60/75 patients (80.0%) in the glasdegib + LDAC arm and 28/36 patients (77.8%) in the LDAC alone arm. The most frequently reported (> 15% of patients) serious AEs were febrile neutropenia (glasdegib + LDAC, 28.0%; LDAC alone, 16.7%) and pneumonia (glasdegib + LDAC, 21.3%; LDAC alone, 19.4%). In total, 37.3% of patients who received glasdegib + LDAC and 47.2% receiving LDAC alone permanently discontinued treatment due to AEs. There were no discontinuations resulting from Hedgehog-inhibitor-class–effect AEs (muscle spasms, ageusia/dysgeusia, alopecia, weight loss or asthenia). The main cause of death in both treatment arms was disease progression.

### Biomarkers

Eighty-three patients had baseline mutational analyses of bone marrow and/or peripheral blood: 41 with de novo AML (glasdegib + LDAC, *n* = 30; LDAC alone, *n* = 11) and 42 with secondary AML (glasdegib + LDAC, *n* = 28; LDAC alone, *n* = 14). For each mutation, only a relatively small number of patients were evaluable. Thus, the effect of baseline mutations on OS was determined in genes with a mutational frequency of ≥ 5 mutations (Online Resource, Table S5). In the de novo AML subgroup of patients receiving glasdegib + LDAC, there was no statistical (*p* > 0.05) difference in OS for the five genes with a mutational frequency of > 5 mutations: *DNMT3A*, combined *FLT3* (*ITD* and *TKD*), *IDH1*, *IDH2I*, and *RUNX1*. No genes in the LDAC alone arm had ≥ 5 mutations. For patients in the secondary AML subgroup, ≥ 5 mutations were reported in three genes in the glasdegib + LDAC arm (*DNMT3A*, *RUNX1*, and *TET2*) and one gene in the LDAC alone arm (*TET2*). Of the three genes in the glasdegib + LDAC arm, OS correlated only with mutations in *DNMT3A* (HR 4.35; 95% CI 1.40–13.53; *p =* 0.0056*),* where patients with secondary AML and mutated *DNMT3A* had a shorter OS than patients with wild-type *DNMT3A*; the mutational status of *DNMT3A* did not influence OS in patients with de novo AML. *TET2* was the only mutation frequent enough to allow for a comparison between the two treatment arms, and no correlation between OS and mutational status was observed (*p* > 0.05).

## Discussion

This long-term (> 40 months) analysis of the BRIGHT 1003 AML study confirms and expands upon previously reported results by demonstrating a statistically significant improvement in survival among patients with AML receiving glasdegib + LDAC versus LDAC alone [18, 19]. The survival benefit of glasdegib was observed across subgroups defined by baseline characteristic. Post-hoc subgroup analyses demonstrated improved OS with glasdegib + LDAC versus LDAC alone in both patients with de novo AML and secondary AML. Furthermore, improvement was consistent across groups stratified by cytogenetic risk, except in patients with de novo AML plus poor-risk cytogenetics where the small sample size precluded meaningful comparisons.

In the exploratory post-hoc analysis, the survival benefit with glasdegib + LDAC (vs LDAC alone) was most pronounced in patients with secondary AML (HR 0.287; 95% CI 0.151–0.548; *p* < 0.0001). Among patients receiving glasdegib + LDAC, observed CR rates were similar whether patients were diagnosed with de novo (18.4%) or secondary AML (20.0%). As previously demonstrated in the overall population in this study, the addition of glasdegib to LDAC significantly improved OS, even among patients who did not achieve CR [23]. Together, these data suggest that patients may benefit from receiving glasdegib in the absence of remission, especially in those with secondary AML.

Secondary AML is biologically distinct from de novo AML. Unique biological features such as subclonal heterogeneity, upregulation of anti-apoptotic proteins, and multidrug resistance make secondary AML treatment challenging [16]. Low blast count AML (20–30% blasts) is genetically and clinically similar to high-risk MDS, and the response to HMAs and prognosis is comparable between these subgroups [24, 25]. In view of these favorable responses to glasdegib + LDAC, this approach is being investigated in patients with higher-risk MDS [26]. In the primary analysis of the BRIGHT 1003 AML study, a 22.8% reduction in the risk of death for glasdegib + LDAC relative to LDAC alone was observed in a small subset of patients with MDS [18]. The impact of glasdegib + azacitidine in patients with previously untreated MDS is also currently under evaluation in a single-arm phase II trial (ClinicalTrials.gov identifier: NCT02367456).

Preliminary signs of clinical efficacy were evident across patients with diverse mutational profiles; however, small patient numbers prevent firm conclusions from being drawn based on mutational status for some genes in this analysis. In the de novo AML subgroup, there was no statistical difference in OS according to the mutational status of *DNMT3A*, combined *FLT3*, *IDH1*, *IDH2*, and *RUNX1* genes in the glasdegib + LDAC arm. For patients in the secondary AML subgroup, there was no statistical difference in OS according to mutational status of *RUNX1* and *TET2* genes in the glasdegib + LDAC arm; however, there was a negative correlation with mutations in *DNMT3A* (*p =* 0.0056) compared with patients with wild-type *DNMT3A*. *DNMT3A* mutations were associated with a short OS in both the glasdegib + LDAC and LDAC alone arms (3.1 and 1.9 months, respectively). *DNMT3A* is a DNA methyltransferase that catalyzes the transfer of a methyl group onto the 5′-position of cytosine of CpG dinucleotides. Mutations in *DNMT3A* are thought to play a pivotal role in the initiation of clonal hematopoiesis and provide a fertile ground for AML transformation. The prognostic implications of *DNMT3A* mutations are inconclusive, with a number of studies suggesting that mutations of *DNMT3A* confer a poor prognosis [27]. In this study, mutational status of *DNMT3A* correlated with OS during glasdegib + LDAC treatment in patients with secondary AML, but not in patients with de novo AML.

Long-term follow-up confirmed that treatment with glasdegib + LDAC was associated with an acceptable safety profile in patients with AML, with little additional toxicity seen with the combination of glasdegib + LDAC versus LDAC alone. No patients discontinued treatment because of AEs associated with inhibition of the Hedgehog signaling pathway in normal tissues (e.g., muscle spasms, ageusia/dysgeusia, alopecia, weight loss, and asthenia)*.* The smoothened antagonists vismodegib and sonidegib are approved for the treatment of advanced basal cell carcinoma; however, their efficacy has yet to be demonstrated in the treatment of AML [28–30]. Although the on-target profile of glasdegib is consistent with both agents, the rate and severity of AEs are lower with glasdegib treatment. The most commonly occurring AEs with vismodegib and sonidegib included alopecia, dysgeusia, and muscle spasms. The differences between the safety profiles may in part be explained by the shorter elimination half-life of glasdegib (sonidegib, 28 days; vismodegib, 4 days; glasdegib, 17.4 h) [28, 29, 31].

As the Hedgehog signaling pathway is not essential for adult hematopoietic stem cell function, targeting leukemic stem cells with glasdegib may allow a reduction in tumor burden while maintaining normal hematopoiesis [32, 33]. Patients receiving glasdegib + LDAC had improved cell-lineage recovery data, suggesting that the treatment combination provides clinical benefit by reducing the risk of cytopenias in this patient population. More patients receiving glasdegib + LDAC were transfusion-independent and, when exposure-adjusted, LDAC alone patients required transfusions twice as often. These results were consistent in both patients with de novo and secondary AML.

Until recently, the general therapeutic strategy for patients with AML who are ineligible for intensive chemotherapy was treatment with LDAC or HMAs. A meta-analysis including patients unable to receive intensive chemotherapy, because of comorbidities or older age (≥ 75 years), demonstrated that the median OS for patients receiving treatment with azacitidine, decitabine, or LDAC was 6.3 months [34]. The recent US Food and Drug Administration approvals of glasdegib and venetoclax in combination with standard-of-care therapies has provided new therapeutic options for this patient population [17, 18, 35]. In this study, the combination of glasdegib + LDAC was shown to be most beneficial in patients with secondary AML. Although comparison between trials should be considered with caution due to potential methodologic and other differences, the median OS for patients with secondary AML receiving glasdegib + LDAC compared favorably with previously reported outcomes in patients receiving enasidenib (8.8 months), decitabine (7.1 months), and venetoclax + LDAC (4.0 months) [13, 35, 36]. In addition, the median OS with glasdegib + LDAC was comparable to that in patients with secondary or therapy-related AML who were eligible for intensive chemotherapy and who received treatment with CPX-351 (9.6 months) [10]. Considering the specified criteria to select patients who were ineligible for intensive chemotherapy in our study, with a median age nearly a decade older than in the CPX-351 study, and more than half of patients with an ECOG PS of 2 (compared with < 10% in the CPX-351 study), as well as significant comorbidities (e.g., cardiac disease in > 50%), this analysis suggests that glasdegib + LDAC is a valuable alternative for these patients.

In conclusion, long-term analysis of the BRIGHT AML 1003 study continued to show superior OS with glasdegib + LDAC (vs LDAC alone) in patients with AML who were ineligible for intensive chemotherapy. The clinical benefit with glasdegib + LDAC was most pronounced in patients with secondary AML, with a statistically significant and clinically meaningful improvement in OS compared with LDAC alone. The combination of glasdegib + LDAC represents a promising treatment strategy for patients with secondary AML who are ineligible for intensive chemotherapy. Glasdegib is currently in phase III clinical development (BRIGHT AML 1019; ClinicalTrials.gov identifier: NCT03416179) for patients with AML in combination with azacitidine or 7 + 3 intensive chemotherapy.

## Supplementary information

ESM 1 (PDF 1.35 mb).

## Data Availability

Upon request, and subject to certain criteria, conditions, and exceptions (see https://www.pfizer.com/science/clinical-trials/trial-data-and-results for more information), Pfizer will provide access to individual de-identified participant data from Pfizer-sponsored global interventional clinical studies conducted for medicines, vaccines, and medical devices (1) for indications that have been approved in the USA and/or EU, or (2) in programs that have been terminated (i.e., development for all indications has been discontinued). Pfizer will also consider requests for the protocol, data dictionary, and statistical analysis plan. Data may be requested from Pfizer trials 24 months after study completion. The de-identified participant data will be made available to researchers whose proposals meet the research criteria and other conditions, and for which an exception does not apply, via a secure portal. To gain access, data requestors must enter into a data access agreement with Pfizer.
