# Correction to: Clinical benefit of glasdegib plus low-dose cytarabine in patients with de novo and secondary acute myeloid leukemia: long-term analysis of a phase II randomized trial

**DOI:** 10.1007/s00277-021-04545-5

**Published:** 2021-05-12

**Authors:** Michael Heuser, B. Douglas Smith, Walter Fiedler, Mikkael A. Sekeres, Pau Montesinos, Brian Leber, Akil Merchant, Cristina Papayannidis, José A. Pérez-Simón, Caroline J. Hoang, Thomas O’Brien, Weidong Wendy Ma, Mirjana Zeremski, Ashleigh O’Connell, Geoffrey Chan, Jorge E. Cortes

**Affiliations:** 1 Department of Hematology, Hemostasis, Oncology and Stem Cell Transplantation, Hannover Medical School, Carl-Neuberg-Str. 1, 30625 Hannover, Germany; 2 Johns Hopkins Sidney Kimmel Comprehensive Cancer Center, Baltimore, MD USA; 3 Department of Hematology and Oncology, University Hospital Hamburg-Eppendorf, Hamburg, Germany; 4 Division of Hematology, Sylvester Comprehensive Cancer Center, University of Miami, Miami, FL USA; 5 Hospital Universitari I Politècnic La Fe, Valencia, Spain; 6 CIBERONC, Instituto Carlos III, Madrid, Spain; 7 Juravinski Hospital At Hamilton Health Sciences, Hamilton, ON Canada; 8 Samuel Oschin Comprehensive Cancer Institute, Cedars-Sinai Medical Center, Los Angeles, CA USA; 9 IRCCS Azienda Ospedaliero-Universitaria Di Bologna, Bologna, Italy; 10 Hospital Universitario Virgen del Rocío, Instituto de Biomedicina (IbiS)/CSIC/CIBERONC), Universidad de Sevilla, Seville, Spain; 11 Pfizer Inc, New York, NY USA; 12 University of Texas MD Anderson Cancer Center, Houston, TX USA; 13 Present Address: Georgia Cancer Center, Augusta, GA USA

## Correction to: Annals of Hematology https://doi.org/10.1007/s00277-021-04465-4

Due to an oversight during the preparation of figures for manuscript submission, the numbers of patients at risk were omitted from the bottom of Fig. 1, panel C. These have now been added along the x-axis to ensure that readers have the most complete information regarding the analysis shown.

**Fig. 1:**
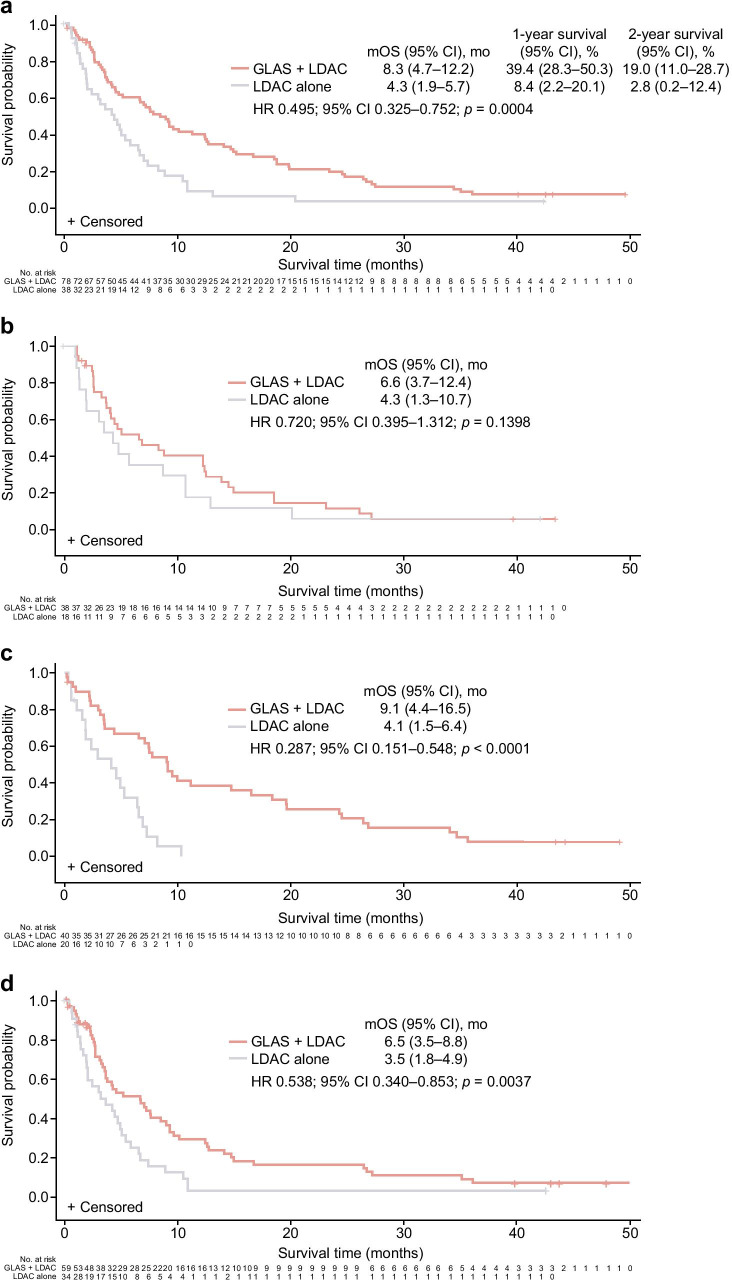
Kaplan–Meier plots of overall survival in the **a** overall population, **b** de novo AML subgroup, **c** secondary AML subgroup, and **d** overall population censoring for patients receiving follow-up HMAs. GLAS, glasdegib; mo, months; mOS, median overall survival

The original article has been corrected.

