# Supplementary material for: Clinical benefit of glasdegib plus low-dose cytarabine in patients with de novo and secondary acute myeloid leukemia: long-term analysis of a phase II randomized trial
Source: Ann Hematol. 2021 Mar 19;100(5):1181–94. doi: 10.1007/s00277-021-04465-4 (PMC8043884; doi:10.1007/s00277-021-04465-4)
Supplement: Supplementary file 1 — (PDF 1.35 mb). [file 277_2021_4465_MOESM1_ESM.docx]

**SUPPLEMENTARY INFORMATION**

**Supplementary Fig. S1** Patient disposition. This study was ongoing at the time of the data cut-off (11 October 2018); the first patient randomization visit took place on 3 January 2014. AE, adverse event; AML, acute myeloid leukemia; GLAS, glasdegib; LDAC, low-dose cytarabine

**
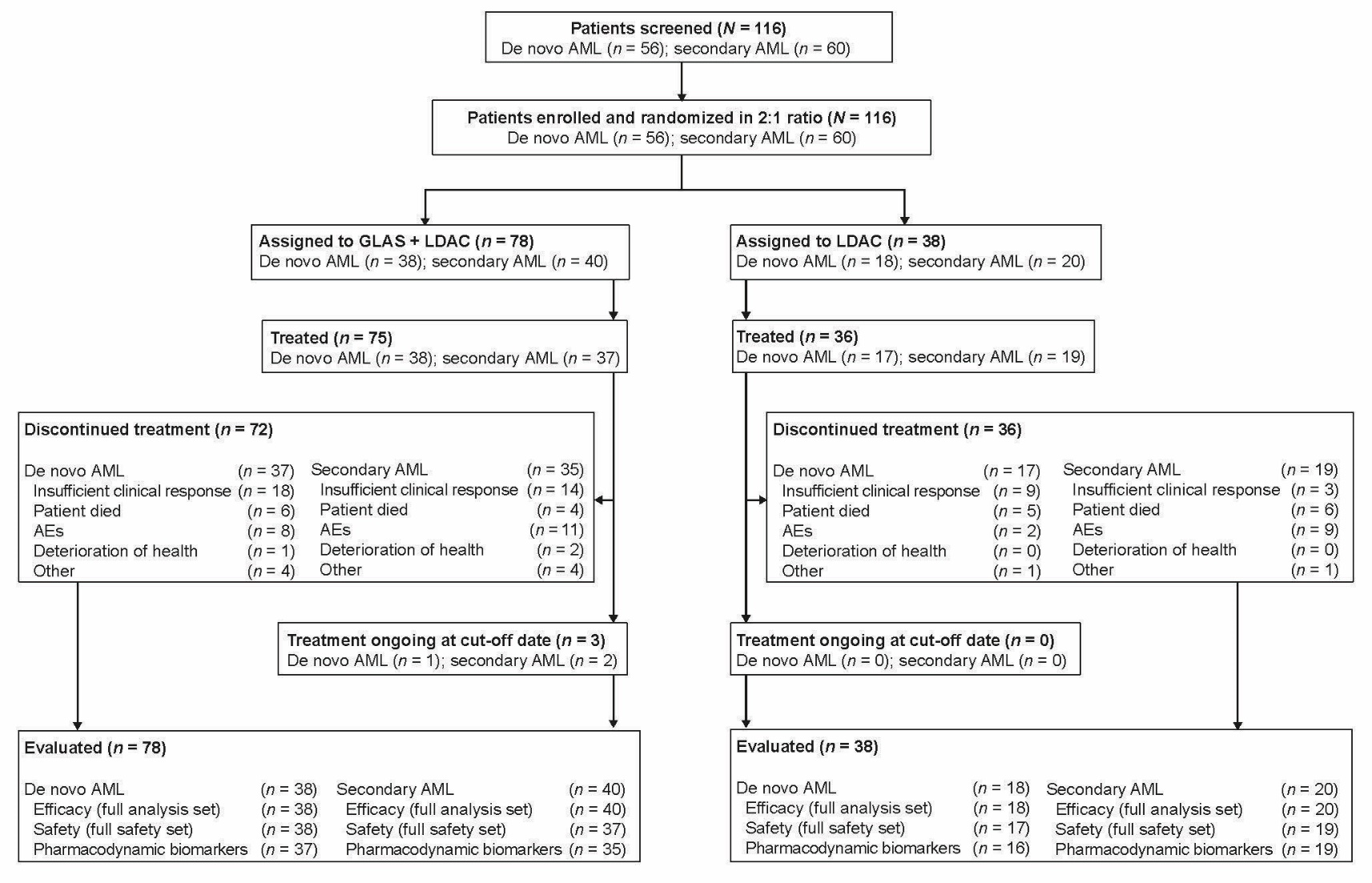
**

**Supplementary Table S1** Patients who received ≥ 1 year of treatment

| Age | Sex | AML diagnosis | ELN risk stratification [22] | Duration of therapy, days | Best response achieved | Duration of CR, days | Overall survival, months | Treatment arm | Reason for treatment discontinuation^a^ |
| --- | --- | --- | --- | --- | --- | --- | --- | --- | --- |
| 81 | M | Secondary AML | Intermediate I | 387 | MR | N/A | 19.5 | Glasdegib + LDAC | Insufficient clinical response |
| 79 | M | de novo | Intermediate II | 412 | CR/CRi | N/A | 18.5 | Glasdegib + LDAC | Insufficient clinical response |
| 69 | M | Secondary AML | Intermediate II | 434 | MR | N/A | 14.7 | Glasdegib + LDAC | Adverse event: pneumonia |
| 72 | F | Secondary AML | Intermediate I | 520 | CR | 457 | 24.4 | Glasdegib + LDAC | Insufficient clinical response |
| 65 | F | Secondary AML | Intermediate II | 540 | CR | 489 | 34.6 | Glasdegib + LDAC | Adverse event: subarachnoid hemorrhage^b,c^ |
| 72 | M | Secondary AML | Intermediate I | 651 | CR | 302 | 34.0 | Glasdegib + LDAC | Insufficient clinical response |
| 74 | F | Secondary AML | Intermediate I | 694 | CR | 610 | 26.8 | Glasdegib + LDAC | Insufficient clinical response |
| 74 | M | de novo | Intermediate II | 707 | CR | 533 | 26.1 | Glasdegib + LDAC | Adverse event: stroke^c^ |
| 76 | M | Secondary AML | Intermediate I | 788 | CR/CRi | N/A | 26.3 | Glasdegib + LDAC | Adverse event: pneumonia |
| 82 | M | Secondary AML | Intermediate I | 972 | CR | 876 | 35.6 | Glasdegib + LDAC | Adverse event: increased creatinine |
| 81 | M | de novo | Intermediate I | 1206 | PR | N/A | 39.7 | Glasdegib + LDAC | N/A |
| 73 | M | Secondary AML | Intermediate II | 1345 | CR | 1262 | 44.2 | Glasdegib + LDAC | N/A |
| 78 | M | Secondary AML | Favorable | 1492 | CR | 574 | 49.1 | Glasdegib + LDAC | N/A |

AML, acute myeloid leukemia; CR, complete remission; CRi, complete remission with incomplete hematologic response; ELN, European LeukemiaNet; LDAC, low-dose cytarabine; MR, minor response; N/A, not applicable; PR, partial response

^a^As determined by investigator assessment

^b^Patient discontinued treatment in CR

^c^Discontinuation was due to an adverse event unrelated to the study treatment or the disease under study

**Supplementary Fig. S2** Forest plot of overall survival for patients with de novo AML. AML, acute myeloid leukemia; CI, confidence interval; ECOG PS, Eastern Cooperative Oncology Group performance status; GLAS, glasdegib; HR, hazard ratio; LDAC, low-dose cytarabine; NE, not evaluable

**
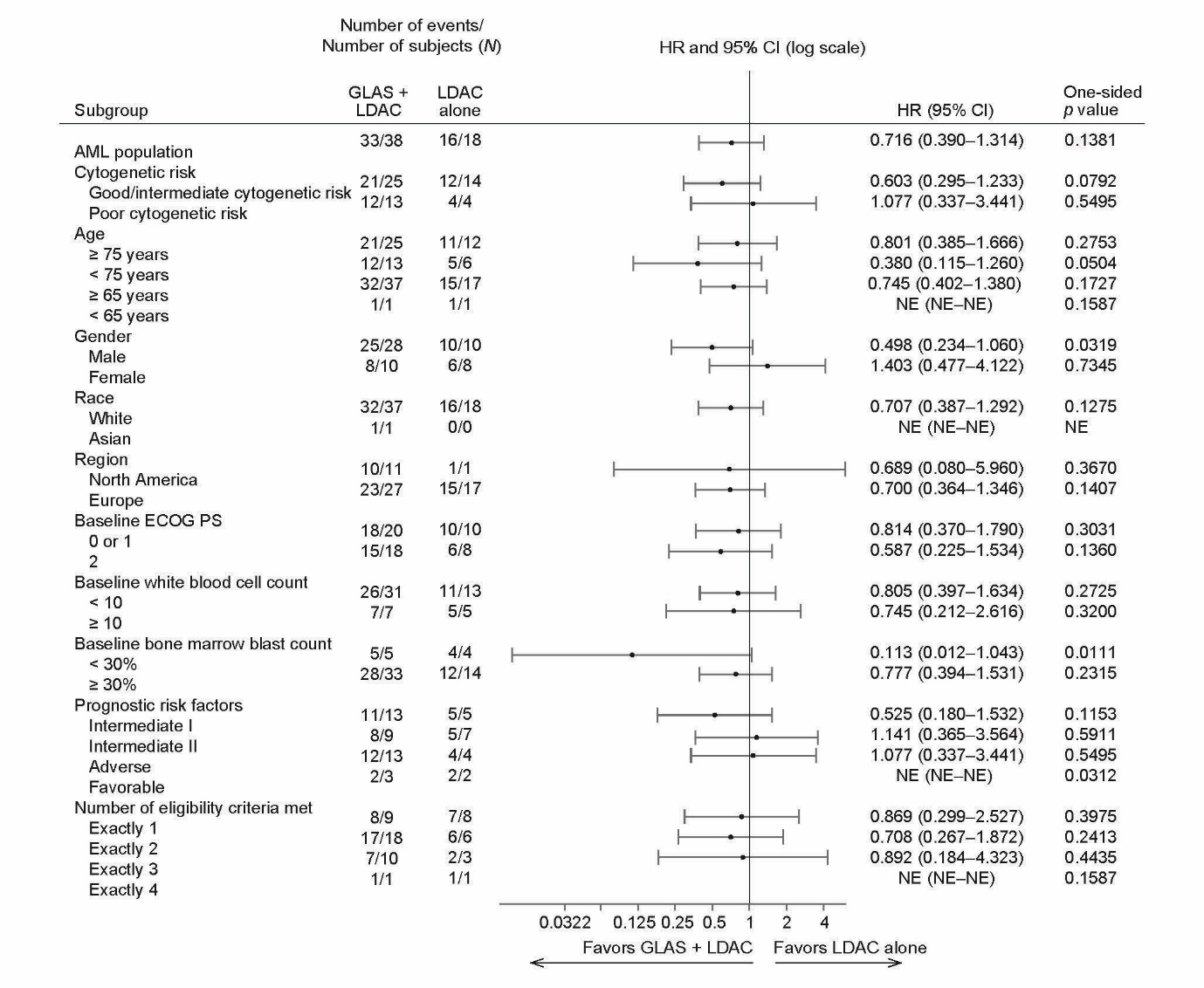
**

**Supplementary Fig. S3** Forest plot of overall survival for patients with secondary AML. AML, acute myeloid leukemia; CI, confidence interval; ECOG PS, Eastern Cooperative Oncology Group performance status; GLAS, glasdegib; HR, hazard ratio; LDAC, low-dose cytarabine; MDS, myelodysplastic syndrome; NE, not evaluable

**
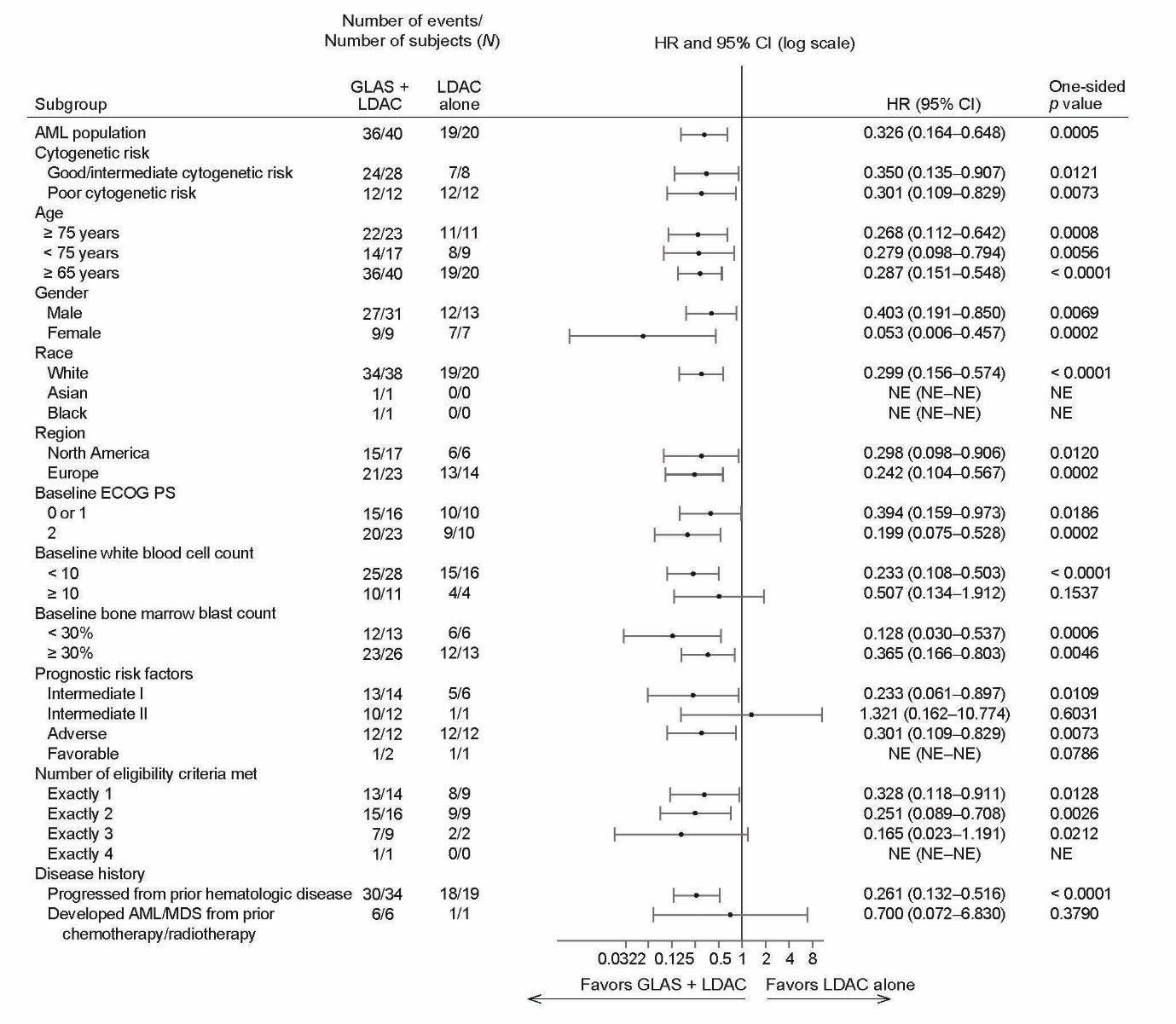
**

**Supplementary Fig. S4** Cumulative incidence of ANC recovery (overall population). ANC, absolute neutrophil count; GLAS, glasdegib; LDAC, low-dose cytarabine


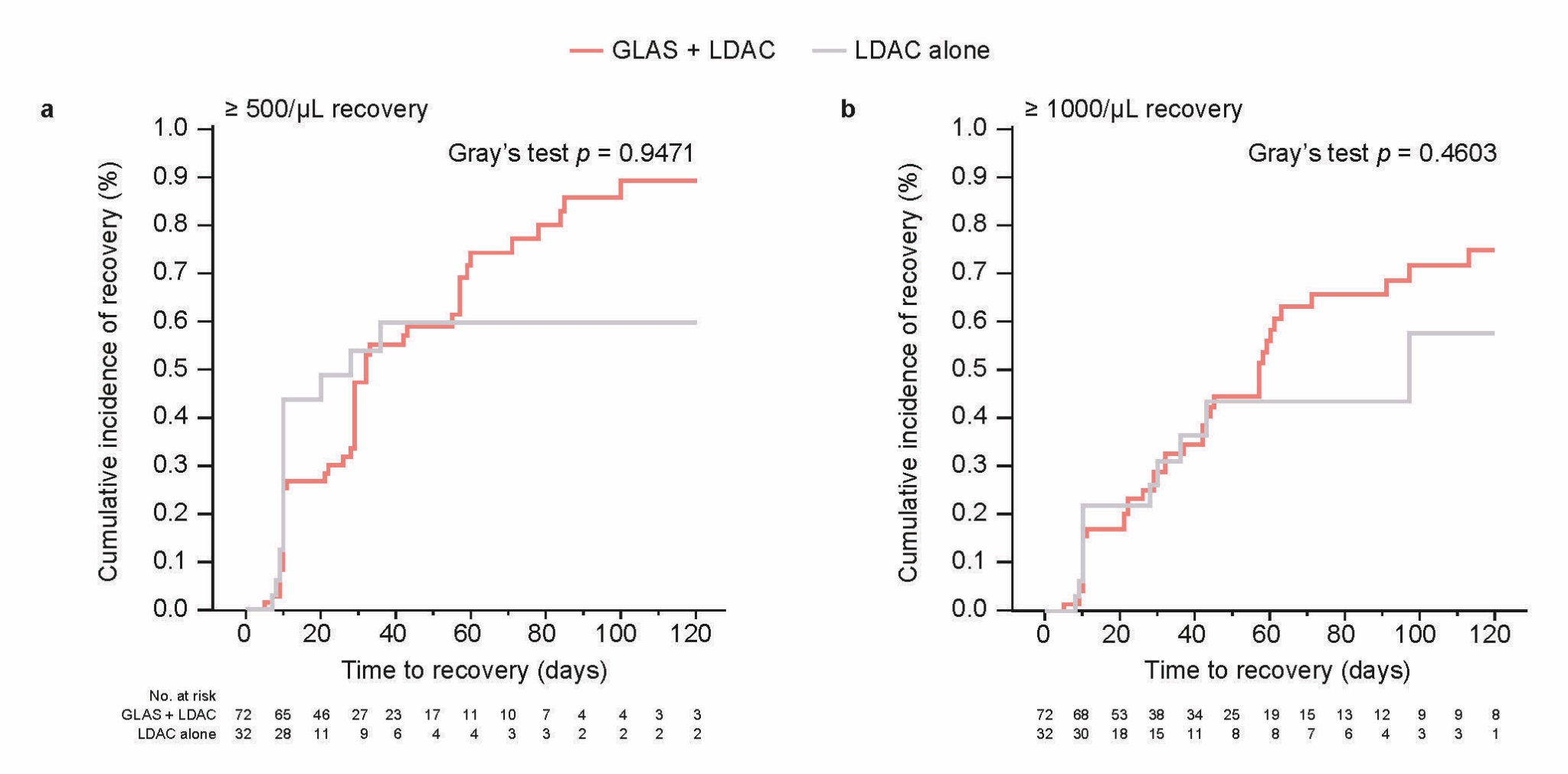


**Supplementary Table S2** Recovery of ANC, hemoglobin, and platelets, and rates of transfusions, by diagnosis of AML

|  | de novo AML | | | | | Secondary AML | | | | | |  |
| --- | --- | --- | --- | --- | --- | --- | --- | --- | --- | --- | --- | --- |
|  | Glasdegib + LDAC | LDAC alone | | Glasdegib + LDAC | LDAC alone | | Glasdegib + LDAC | LDAC alone | | Glasdegib + LDAC | LDAC alone |  |
|  | *n* = 37 | *n* = 16 | | *n* = 37 | *n* = 16 | | *n* = 35 | *n* = 16 | | *n* = 35 | *n* = 16 |  |
| **ANC** | **≥ 1000/µL** | | | **≥ 500/µL** | | | **≥ 1000/µL** | | | **≥ 500/µL** | |  |
| All patients with recovery, *n* (%) | 22 (59.5) | 10 (62.5) | | 27 (73.0) | 11 (68.8) | | 27 (77.1) | 10 (62.5) | | 31 (88.6) | 13 (81.3) |  |
| Recovery at ≥ 2 consecutive visits, *n* (%) | 20 (54.1) | 7 (43.8) | | 22 (59.5) | 8 (50.0) | | 20 (57.1) | 5 (31.3) | | 25 (71.4) | 9 (56.3) |  |
| Baseline ANC < threshold, *n* (%)^a^ | 9 (24.3) | 3 (18.8) | | 8 (21.6) | 1 (6.3) | | 13 (37.1) | 2 (12.5) | | 10 (28.6) | 0 |  |
| Median time to recovery, days (range) | 24 (10–72) | 13 (10–70) | | 14 (10–143) | 11 (10–119) | | 28 (7–114) | 12 (8–29) | | 21 (3–90) | 11 (8–57) |  |
| Achieved recovery during cycle 2/1, *n* (%)^b^ | 13 (35.1) | 6 (37.5) | | 24 (64.9) | 10 (62.5) | | 17 (48.6) | 4 (25.0) | | 25 (71.4) | 12 (75.0) |  |
| **Hemoglobin** | **≥ 10 g/dL** | | | **≥ 9 g/dL** | | | **≥ 10 g/dL** | | | **≥ 9 g/dL** | |  |
| All patients with recovery, *n* (%) | 20 (54.1) | 9 (56.3) | | 34 (91.9) | 11 (69.8) | | 23 (65.7) | 9 (56.3) | | 30 (85.7) | 11 (68.8) |  |
| Recovery at ≥ 2 consecutive visits, *n* (%) | 9 (24.3) | 3 (18.8) | | 21 (56.8) | 7 (43.8) | | 14 (40.0) | 4 (25.0) | | 23 (65.7) | 6 (37.5) |  |
| Baseline hemoglobin < threshold, *n* (%)^a^ | 5 (13.5) | 2 (12.5) | | 11 (29.7) | 1 (6.3) | | 14 (40.0) | 2 (12.5) | | 13 (37.1) | 1 (6.3) |  |
| Median time to recovery, days (range) | 26 (9–129) | 36 (11–140) | | 14 (4–172) | 21 (3–85) | | 22 (6–95) | 31 (9–58) | | 14 (6–43) | 23 (2–33) |  |
| Achieved recovery during cycle 1, *n* (%) | 12 (32.4) | 5 (31.3) | | 28 (75.7) | 9 (56.3) | | 19 (54.3) | 6 (37.5) | | 29 (82.9) | 11 (68.8) |  |
| **Platelets** | **≥ 100,000/µL** | | | **≥ 50,000/µL** | | | **≥ 100,000/µL** | | | **≥ 50,000/µL** | |  |
| All patients with recovery, *n* (%) | 18 (48.6) | 4 (25.0) | | 29 (78.4) | 7 (43.8) | | 18 (51.4) | 3 (18.8) | | 20 (57.1) | 6 (37.5) |  |
| Recovery at ≥ 2 consecutive visits, *n* (%) | 15 (40.5) | 3 (18.8) | | 21 (56.8) | 5 (31.3) | | 15 (42.9) | 1 (6.3) | | 17 (48.6) | 3 (18.8) |  |
| Baseline platelets < threshold, *n* (%)^a^ | 9 (24.3) | 2 (12.5) | | 6 (16.2) | 3 (18.8) | | 11 (31.4) | 0 | | 9 (25.7) | 0 |  |
| Median time to recovery, days (range) | 29 (6–143) | 27 (13–56) | | 25 (4–113) | 22 (6–119) | | 31 (21–171) | 26 (2–29) | | 29 (10–141) | 30 (2–44) |  |
| Achieved recovery during cycle 1, *n* (%) | 12 (32.4) | 3 (18.8) | | 23 (62.2) | 6 (37.5) | | 12 (34.3) | 3 (18.8) | | 17 (48.6) | 5 (31.3) |  |
| Transfusion rates | Glasdegib + LDAC  *n* = 38 | | LDAC alone  *n* = 17 | | | Glasdegib + LDAC  *n* = 37 | | | LDAC alone  *n* = 19 | | |  |
| Proportion independent, *n* (%)^c^ | | | | | |  | | | | | | |
| No transfusions | 11 (28.9) | | 2 (11.8) | | | 11 (29.7) | | | 0 | | |  |
| PRBC transfusions | 12 (31.6) | | 2 (11.8) | | | 13 (35.1) | | | 1 (5.3) | | |  |
| Platelet transfusions | 17 (44.7) | | 4 (23.5) | | | 15 (40.5) | | | 0 | | |  |
| Exposure-adjusted rate | | | | | |  | | | | | | |
| Any transfusion | 0.0647 | | 0.1199 | | | 0.0724 | | | 0.1980 | | |  |
| PRBC transfusion | 0.0382 | | 0.0637 | | | 0.0446 | | | 0.0970 | | |  |
| Platelet transfusion | 0.0265 | | 0.0562 | | | 0.0278 | | | 0.1010 | | |  |

AML, acute myeloid leukemia; ANC, absolute neutrophil count; LDAC, low-dose cytarabine; PRBC, packed red blood cell

^a^Requires measurement at ≥ 2 consecutive visits

^b^Cycle 2 for ANC ≥ 1000/µL and cycle 1 for ANC ≥ 500/µL

^c^Required no PRBC or platelet transfusions for ≥ 8 weeks

**Supplementary Table S3** Safety overview during the first 90 days and after 90 days

| *N* (%) | Overall population | | de novo AML | | Secondary AML | |
| --- | --- | --- | --- | --- | --- | --- |
|  | Glasdegib +  LDAC | LDAC alone | Glasdegib +  LDAC | LDAC alone | Glasdegib +  LDAC | LDAC alone |
| **During the first 90 days** | ***n* = 75** | ***n* = 36** | ***n* = 38** | ***n* = 17** | ***n* = 37** | ***n* = 19** |
| Any AE | 74 (98.7) | 36 (100.0) | 38 (100.0) | 17 (100.0) | 36 (97.3) | 19 (100.0) |
| Any serious AEs | 49 (65.3) | 26 (72.2) | 21 (55.3) | 10 (58.8) | 28 (75.7) | 16 (84.3) |
| Grade 3 or 4 AEs | 63 (84.0) | 33 (91.7) | 32 (84.2) | 14 (82.4) | 31 (83.8) | 19 (100.0) |
| Any grade 5 AEs | 12 (16.0) | 13 (36.1) | 7 (18.4) | 6 (35.3) | 5 (13.5) | 7 (36.8) |
| Discontinued study due to AEs | 17 (22.7) | 12 (33.3) | 9 (23.7) | 5 (29.4) | 8 (21.6) | 7 (36.8) |
| Glasdegib dose reduced due to AEs | 8 (10.7) | N/A | 2 (5.3) | N/A | 6 (16.2) | N/A |
| Glasdegib interrupted due to AEs | 38 (50.7) | N/A | 18 (47.4) | N/A | 20 (54.1) | N/A |
| LDAC dose reduced due to AEs | 5 (6.7) | 0 | 2 (5.3) | 0 | 3 (8.1) | 0 |
| LDAC therapy interrupted due to AEs | 20 (26.7) | 9 (25.0) | 10 (26.3) | 4 (23.5) | 10 (27.0) | 5 (26.3) |
| **After 90 days** | ***n* = 43** | ***n* = 14** | ***n* = 21** | ***n* = 7** | ***n* = 22** | ***n* = 7** |
| Any AEs | 36 (83.7) | 10 (71.4) | 16 (76.2) | 5 (71.4) | 20 (90.9) | 5 (71.4) |
| Any serious AEs | 22 (51.2) | 7 (50.0) | 10 (47.6) | 3 (42.9) | 12 (54.5) | 4 (57.1) |
| Grade 3 or 4 AEs | 30 (69.8) | 8 (57.1) | 12 (57.1) | 4 (57.1) | 18 (81.8) | 4 (57.1) |
| Any grade 5 AEs | 10 (23.3) | 3 (21.4) | 5 (23.8) | 3 (42.9) | 5 (22.7) | 0 |
| Discontinued study due to AEs | 11 (25.6) | 5 (35.7) | 3 (14.3) | 1 (14.3) | 8 (36.4) | 4 (57.1) |
| Glasdegib dose reduced due to AEs | 5 (11.6) | N/A | 3 (14.3) | N/A | 2 (9.1) | N/A |
| Glasdegib interrupted due to AEs | 17 (39.5) | N/A | 7 (33.3) | N/A | 10 (45.5) | N/A |
| LDAC dose reduced due to AEs | 7 (16.3) | 0 | 2 (9.5) | 0 | 5 (22.7) | 0 |
| LDAC interrupted due to AEs | 15 (34.9) | 4 (28.6) | 6 (28.6) | 3 (42.9) | 9 (40.9) | 1 (14.3) |

AE, adverse event; AML, acute myeloid leukemia; LDAC, low-dose cytarabine; N/A, not applicable

**Supplementary Table S4** Treatment-emergent all-causality AEs occurring in ≥ 25% of patients (de novo and secondary AML) in any treatment arm during the first 90 days and after 90 days of therapy

| MedDRA preferred term, *n* (%) | de novo AML | | Secondary AML | |
| --- | --- | --- | --- | --- |
|  | Glasdegib +  LDAC | LDAC alone | Glasdegib +  LDAC | LDAC alone |
| **During the first 90 days** | ***n* = 38** | ***n* = 17** | ***n* = 37** | ***n* = 19** |
| Any AEs | 38 (100.0) | 17 (100.0) | 36 (97.3) | 19 (100.0) |
| Anemia | 16 (42.1) | 8 (47.1) | 17 (45.9) | 7 (36.8) |
| Nausea | 13 (34.2) | 0 | 9 (24.3) | 4 (21.1) |
| Thrombocytopenia | 12 (31.6) | 4 (23.5) | 11 (29.7) | 5 (26.3) |
| Dysgeusia | 10 (26.3) | 1 (5.9) | 5 (13.5) | 0 |
| Febrile neutropenia | 10 (26.3) | 3 (17.6) | 13 (35.1) | 5 (26.3) |
| Diarrhea | 5 (13.2) | 5 (29.4) | 8 (21.6) | 4 (21.1) |
| Fatigue | 9 (23.7) | 1 (5.9) | 10 (27.0) | 5 (26.3) |
| Decreased appetite | 6 (15.8) | 1 (5.9) | 9 (24.3) | 2 (10.5) |
| Dyspnea | 6 (15.8) | 4 (23.5) | 7 (18.9) | 5 (26.3) |
| Pyrexia | 6 (15.8) | 3 (17.6) | 9 (24.3) | 5 (26.3) |
| **After 90 days** | ***n* = 21** | ***n* = 7** | ***n* = 22** | ***n* = 7** |
| Any AEs | 16 (76.2) | 5 (71.4) | 20 (90.9) | 5 (71.4) |
| Muscle spasms | 7 (33.3) | 0 | 3 (13.6) | 0 |
| Decreased appetite | 6 (28.6) | 1 (14.3) | 7 (31.8) | 2 (28.6) |
| Anemia | 4 (19.0) | 2 (28.6) | 9 (40.9) | 1 (14.3) |
| Diarrhea | 5 (23.8) | 1 (14.3) | 9 (40.9) | 0 |
| Pneumonia | 1 (4.8) | 0 | 7 (31.8) | 1 (14.3) |
| Pyrexia | 3 (14.3) | 0 | 7 (31.8) | 1 (14.3) |
| Renal failure | 0 | 0 | 1 (4.5) | 2 (28.6) |

AE, adverse event; AML, acute myeloid leukemia; LDAC, low-dose cytarabine; MedDRA, Medical Dictionary for Regulatory Activities

**Supplementary Table S5** Baseline gene mutation status and OS in patients with de novo and secondary AML

|  | | de novo AML | | | | Secondary AML | | | |
| --- | --- | --- | --- | --- | --- | --- | --- | --- | --- |
|  |  | Glasdegib + LDAC  *n* = 30 | | LDAC alone  *n* = 11 | | Glasdegib + LDAC  *n* = 28 | | LDAC alone  *n* = 14 | |
| Baseline gene mutation | Mutational status | *n* (%) | Median  OS, months (95% CI) | *n* (%) | Median  OS, months (95% CI) | *n* (%) | Median  OS, months (95% CI) | *n* (%) | Median  OS, months (95% CI) |
| *CEBPA* | Mutated  Non-mutated | 4 (13.3)  26 (86.7) | 4.9 (1.2–12.5)  5.0 (3.6–12.3) | 1 (9.1)  10 (90.9) | 1. (NE–NE)   4.1 (1.3–10.7) | 4 (14.3)  24 (85.7) | 2.1 (0.2–34.6)  9.1 (6.5–18.3) | 2 (14.3)  12 (85.7) | 6.3 (4.5–8.1)  2.6 (0.6–5.3) |
| *DNMT3A* | Mutated  Non-mutated | 10 (33.3)  20 (66.7) | 5.5 (2.4–12.2)  5.0 (2.6–12.5) | 2 (18.2)  9 (81.8) | 6.0 (1.3–10.7)  3.5 (1.0–12.9) | 5 (17.9)  23 (82.1) | 3.1 (0.2–9.0)  12.3 (3.5–24.2) | 4 (28.6)  10 (71.4) | 1.9 (0.6–6.4)  4.3 (0.3–5.3) |
| *FLT3*^a^ | Mutated  Non-mutated | 3 (10.0)  27 (90.0) | 3.3 (2.5–6.6)  5.0 (3.7–12.5) | 0  11 (100.0) | –  3.5 (1.3–10.7) | 2 (7.1)  26 (92.9) | 2.8 (2.1–3.5)  9.1 (6.5–19.6) | 0  14 (100.0) | –  3.5 (1.5–5.3) |
| *FLT3 ITD*^b^ | Mutated  Non-mutated | 2 (6.7)  25 (83.3) | 10.5 (2.4–18.5)  4.7 (3.6–8.8) | 1 (9.1)  9 (81.8) | 1. (NE–NE)   4.8 (1.3–12.9) | 1 (3.6)  26 (92.9) | 6.5 (NE–NE)  9.1 (3.5–19.6) | 1 (7.1)  13 (92.9) | 5.3 (NE–NE)  2.9 (1.5–4.9) |
| *Combined*  *FLT3* | Mutated  Non-mutated | 5 (16.7)  22 (73.3) | 3.3 (2.4–18.5)  5.0 (3.7–12.3) | 1 (9.1)  9 (81.8) | 1.0 (NE–NE)  4.8 (1.3–12.9) | 3 (10.7)  24 (85.7) | 3.5 (2.1–6.5)  9.9 (7.1–19.6) | 1 (7.1)  13 (92.9) | 5.3 (NE–NE)  2.9 (1.5–4.9) |
| *IDH1* | Mutated  Non-mutated | 6 (20.0)  24 (80.0) | 7.8 (3.3–27.1)  4.4 (2.6–12.3) | 2 (18.2)  9 (81.8) | 1.6 (1.3–1.9)  4.8 (1.0–12.9) | 2 (7.1)  26 (92.9) | 13.8 (3.1–24.4)  9.1 (3.5–19.6) | 0  14 (100.0) | –  3.5 (1.5–5.3) |
| *IDH2* | Mutated  Non-mutated | 8 (26.7)  22 (73.3) | 5.0 (1.1–23.1)  4.7 (3.6–12.3) | 0  11 (100.0) | –  3.5 (1.3–10.7) | 3 (10.7)  25 (89.3) | 4.6 (2.2–7.1)  9.1 (3.5–19.6) | 4 (28.6)  10 (71.4) | 5.8 (4.5–6.5)  2.1 (0.3–4.1) |
| *KIT* | Mutated  Non-mutated | 1 (3.3)  29 (96.7) | 3.6 (NE–NE)  5.0 (3.7–12.3) | 0  11 (100.0) | –  3.5 (1.3–10.7) | 2 (7.1)  26 (92.9) | 5.4 (3.4–7.4)  9.1 (3.5–19.6) | 0  14 (100.0) | –  3.5 (1.5–5.3) |
| *KRAS* | Mutated  Non-mutated | 0  30 (100.0) | –  5.0 (3.6–12.2) | 0  11 (100.0) | –  3.5 (1.3–10.7) | 2 (7.1)  26 (92.9) | 4.8 (0.7–9.0)  9.1 (3.5–19.6) | 2 (14.3)  12 (85.7) | 7.3 (6.4–8.1)  2.6 (0.6–4.9) |
| *NPM1* | Mutated  Non-mutated | 3 (10.0)  27 (90.0) | 2.6 (2.5–6.6)  5.0 (3.7–12.5) | 1 (9.1)  10 (90.9) | 1. (NE–NE)   4.1 (1.3–10.7) | 2 (7.1)  26 (92.9) | NE (3.0–NE)  9.1 (3.5–18.3) | 0  14 (100.0) | –  3.5 (1.5–5.3) |
| *NRAS* | Mutated  Non-mutated | 3 (10.0)  27 (90.0) | 2.6 (0.9–NE)  5.0 (3.7–12.3) | 0  11 (100.0) | –  3.5 (1.3–10.7) | 2 (7.1)  26 (92.9) | 1.8 (0.7–3.0)  9.1 (6.5–19.6) | 2 (14.3)  12 (85.7) | 7.3 (6.4–8.1)  2.6 (0.6–4.9) |
| *RUNX1* | Mutated  Non-mutated | 14 (46.7)  16 (53.3) | 6.9 (1.2–13.9)  4.7 (3.3–12.2) | 2 (18.2)  9 (81.8) | 7.7 (4.8–10.7)  3.1 (1.0–12.9) | 11 (39.3)  17 (60.7) | 14.7 (2.3–26.3)  9.0 (3.0–18.3) | 4 (28.6)  10 (71.4) | 3.9 (1.5–5.3)  3.2 (0.3–6.4) |
| *TET2* | Mutated  Non-mutated | 4 (13.3)  26 (86.7) | 3.3 (1.1–12.5)  6.6 (3.6–12.3) | 4 (36.4)  7 (63.6) | 7.1 (1.0–12.9)  3.1 (1.3–5.7) | 10 (35.7)  18 (64.3) | 19.0 (0.7–35.6)  7.4 (3.0–14.7) | 5 (35.7)  9 (64.3) | 6.4 (0.6–8.1)  2.3 (0.3–4.5) |
| *WT1* | Mutated  Non-mutated | 2 (6.7)  28 (93.3) | 7.8 (3.3–12.3)  5.0 (3.6–12.2) | 0  11 (100.0) | –  3.5 (1.3–10.7) | 1 (3.6)  27 (96.4) | 6.5 (NE–NE)  9.1 (3.4–18.3) | 1 (7.1)  13 (92.9) | 6.5 (NE–NE)  2.9 (1.5–4.9) |

AML, acute myeloid leukemia; CI, confidence interval; LDAC, low-dose cytarabine; NE, not evaluable; OS, overall survival

Shaded rows indicate genes with a mutation frequency of ≥ 5 mutations

^a^Includes only *FLT3* point mutations

^b^In a secondary assay, an amplicon-based approach was used to further characterize the *FLT3* gene for the presence of an internal tandem duplication mutation

**REFERENCE**

22. Cortes JE, Dombret H, Merchant A, Tauchi T, DiRienzo CG, Sleight B, Zhang X, Leip EP, Shaik N, Bell T, Chan G, Sekeres MA (2019) Glasdegib plus intensive/nonintensive chemotherapy in untreated acute myeloid leukemia: BRIGHT AML 1019 phase III trials. Future Oncol 15(31):3531–3545. https://doi.org/10.2217/fon-2019-0373
